# No patella resurfacing total knee arthroplasty leads to reduction in the thickness of patellar cartilage to less than half within 5 years: a quantitative longitudinal evaluation using MRI

**DOI:** 10.1186/s40634-021-00425-z

**Published:** 2021-11-24

**Authors:** Dai Sato, Masayuki Inoue, Takuro Sasaki, Jun Uchida, Tomohiro Onodera, Eiji Kondo, Norimasa Iwasaki

**Affiliations:** 1 Department of Orthopaedic Surgery, NTT East Japan Sapporo Hospital, Minami-1, Nishi-15, Chuou-ku, Sapporo, Hokkaido 060-0061 Japan; 2 Department of Orthopaedic Surgery, Hokushin Higashi Orthopedic Hospital, Fushiko-5-3-3-2, Higashi-ku, Sapporo, Hokkaido 007-0865 Japan; 3 Department of Orthopaedic Surgery, Hokkaido Orthopaedic Memorial Hospital, Hiragishi-7-13-5-22, Toyohira-ku, Sapporo, Hokkaido 062-0937 Japan; 4 Department of Orthopedic Surgery, Faculty of Medicine and Graduate School of Medicine, Hokkaido University, Kita-15 Nishi-7, Kita-ku, Sapporo, Hokkaido 060-8638 Japan; 5 Centre for Sports Medicine, Hokkaido University Hospital, Kita-14 Nishi-5, Kita-ku, Sapporo, Hokkaido 060-8648 Japan

**Keywords:** Total knee arthroplasty, Non-patellar resurfacing, Cartilage thickness of the patella, Magnetic resonance imaging

## Abstract

**Purpose:**

Patellar resurfacing in total knee arthroplasty (TKA) remains controversial as recent meta-analyses have not shown its clear superiority; however, most authors recommend it because it is associated with less frequent anterior knee pain and need for reoperation. We aimed to clarify the changes in patellar cartilage thickness in no patellar resurfacing TKA using a ceramic femoral component on magnetic resonance imaging (MRI).

**Methods:**

Between 2009 and 2014, 40 consecutive patients (59 knees) were included in this study. All patients underwent TKA using zirconia ceramic femoral implants without patellar resurfacing. Indications for no patellar resurfacing TKA were absence of anterior knee pain, patellar compression pain, and osteoarthritic changes in the patellofemoral joint on plain radiography. The mean postoperative follow-up duration was 81.5 months (range, 25–131 months). Clinical and radiological evaluations were performed preoperatively and 5 years after TKA. Patellar cartilage thickness was evaluated preoperatively and every year for 5 years after TKA using MRI T2-weighted imaging. The patellar cartilage was divided into three regions of interest: medial, central, and lateral. To standardise the variation in patellar thickness among patients, the percent cartilage thickness was calculated.

**Results:**

The implant’s position was appropriate in all cases. Compared to preoperative scores, 5 years postoperatively, the Japanese Orthopedic Association score and Oxford knee score significantly improved from 52.1 to 84.7; mean tilting angle and congruence angle did not change significantly; mean lateral shift ratio significantly increased from 7.1% to 14.6%; cartilage thickness significantly decreased (*P* < 0.05); and the percentage cartilage thickness of the central, medial, and lateral cartilage zones gradually thinned to less than half. Four patients underwent conversion to patellar resurfacing due to anterior knee pain, without loosening the femoral and tibial implants.

**Conclusion:**

The patellar cartilage thickness decreased to less than half its preoperative level within 5 years after no patellar resurfacing TKA; this would led to clinical problems and conversion to patellar resurfacing.

**Level of evidence:**

Level III.

## Introduction

While total knee arthroplasty (TKA) is commonly performed for end-stage osteoarthritis (OA) of the knee, orthopaedic surgeons are still seeking clarification regarding the indications for patellar resurfacing during this procedure [3]. Currently, the decision to perform patellar resurfacing is still largely based on the surgeon’s preference, experience, and training [27]*.* Some surgeons prefer selective non-resurfacing of the patella for patients with OA [3, 29], while others prefer routine patellar resurfacing for more predictable results [30, 31]. The potential risk of patellar fracture leading to patellar resurfacing and the challenge posed while managing the resurfaced patella at revision has led some authors to advocate the non-resurfacing of patella technique during TKA [26].

Recently published meta-analyses have demonstrated no significant advantages of patellar resurfacing over non-resurfacing during primary TKA [6, 25, 26]. However, most authors recommend patellar resurfacing because it is associated with less frequent anterior knee pain (AKP) and the need for reoperation [4, 21, 22]. Furthermore, the concern is whether the patellar cartilage has been affected after non-resurfacing patellar TKA. To address the effect on patellar cartilage, its radiological evaluation has been considered important. However, postoperative imaging of TKA using magnetic resonance imaging (MRI) is difficult due to the susceptibility of implants, which are generally made of cobalt-chrome, to generate artefacts despite recent metal artefact reduction techniques [11, 16]*.* Therefore, further trials using patellofemoral (PF)-specific evaluations are needed to clarify the effect of non-patellar resurfacing during primary TKA on the PF joint. A previous prospective study demonstrated and compared the quantitative changes in the patellar cartilage 1 year after TKA without patellar resurfacing using delayed gadolinium-enhanced MRI of cartilage (dGEMRIC) and T2 mapping at 3.0 T [18]. The authors of the study concluded that clinical scores were not significantly different between the two groups; however, MRI showed that osteoarthritic changes in the patellar cartilage occurred 1 year after TKA. They speculated that the osteoarthritic changes in the patellar cartilage after non-resurfacing patellar TKA could influence future clinical outcomes. We thought that the wear of patellar cartilage could be detected on conventional MRI at 1.5 T in longitudinal observation. In cases of non-patellar resurfacing, details concerning the changes in patellar cartilage quality over time are crucial to decide whether to resurface the patella. Furthermore, it was necessary to clarify the mid-term effects of changes in the patellar cartilage on clinical outcomes.

We hypothesised that patellar cartilage degeneration occurs after TKA using ceramic implants without patellar resurfacing, and patellar cartilage wear after TKA without patellar resurfacing may cause PF joint pain. The aim of this study was to characterise quantitative and longitudinal changes in patellar cartilage thickness over time after non-resurfacing patellar TKA using ceramic implants by using MRI, and to clarify the effect of changes in the patellar cartilage on clinical outcomes.

## Materials and methods

### Study design

This retrospective cohort study was performed between January 2009 and December 2014, and included 40 consecutive patients (59 knees) who underwent TKA using a zirconia ceramic implant (LFA, PS-type Kyocera Company, Japan)*.* Each patient received conservative treatment for at least 3 months. The inclusion criterion was persistent pain due to OA of Kellgren and Lawrence grade 4, while the exclusion criteria included: (1) AKP in daily life; (2) positive patellar compression test; (3) severe OA in the PF joint on plain radiography and MRI; (4) patellar maltracking during TKA surgery; and (5) a history of infection in the knee.

### Patient demographics

The demographic data are presented in Table 1. There were 5 male and 35 female patients with a mean age of 72.2 (range, 60–83 years) at the time of surgery. Overall, all 59 knees were diagnosed with OA of Kellgren and Lawrence grade 4. This study was approved by the NTT Higashi-Nihon Hospital Institutional Committee on Ethics, and all patients provided informed consent for this study (Trial number: 19–00469).

**Table 1:** Patients’ background characteristics

Variable	Frequency
Age (y)	72.2 (7.3)
Male/female (patients)	5/35
Right/left side (knees)	31/28
BMI (kg/m^2^)	25.3 (4.3)

Data are reported as mean (standard deviation)

*BMI* body mass index

### TKA procedure

All the patients were treated by an experienced surgeon (M.I.) at our hospital. Surgery was performed using the medial parapatellar approach with the patient in prone position. The distal femoral cut was performed first to use an intramedullary alignment rod. To obtain restricted kinematic alignment, we set the alignment perpendicular to the mechanical axis. Next, the tibial cut was performed to use the adjustable intramedullary alignment rod, and the tibial component rotation was aligned to the medial third of the tibial tubercle. We used an independent cut (measured resection), and the condylar twist angle (CTA), which was defined as the angle between the epicondylar and posterior condylar lines, was measured in all cases using computed tomography (CT) and MRI before the operation in order to cut the bone accurately. A bone cut, based on the surgical epicondylar line, was performed in every case. We used the cemented low-friction anatomic (LFA, CR-type, Kyocera Company, Japan) type of prosthesis. This implant is made of zirconia ceramics and is designed especially for the Japanese anatomical shape [33]. The osteophytes and synovium around the patella were removed during the operation. Patella tracking was assessed in all cases, and lateral release was not performed in any case.

### Clinical and radiological evaluations

The patients were evaluated using the Japanese Orthopedic Association (JOA) score for osteoarthritic knees, with a total of 100 points: pain and walking = 30 points, pain and stairs up and down = 25 points, range of motion = 35 points, and swelling = 10 points) [32] and the Oxford knee score [5]. Clinical evaluations were performed preoperatively, 1 year, and 5 years after TKA by three orthopaedic surgeons (T.S., J.U., and D.S.). During physical examination, the range of motion and patellar compression pain were assessed. Radiographs were obtained both before and 5 years after TKA. The lateral femorotibial angle was measured on an anteroposterior weight-bearing radiograph of a single leg with the knee joint in extension. The mechanical axis percentages were measured on an anteroposterior radiograph of the entire lower limb taken with a long cassette in the one-leg standing position. The Insall–Salvati index [14] and Caton–Deschamps index were estimated using lateral radiographs. The tilting angle, congruence angle, and lateral shift ratio [28] were determined from plain X-ray images in the skyline view. To evaluate the accurate placement of the femur and tibia implants, radiographic evaluation of the components after TKA was performed according to the Knee Society Grading Scale [7]. To accurately evaluate the bone cut, the CTA was also measured using axial MRI preoperatively and postoperatively.

### MRI evaluation and method of measuring patellar cartilage thickness

All patients underwent MRI evaluations preoperatively and every year for 5 years after TKA surgery. MRI was performed using a 1.5-Tesla whole-body clinical scanner (Ingenia 1.5 T Ambition; Philips Healthcare, Best, The Netherlands). The PF joint of each subject was centred in a microscope coil in the supine position with the knee slightly flexed. Three-plane (axial, coronal, and sagittal) localiser imaging was performed for the PF joint.

**Fig. 1:**
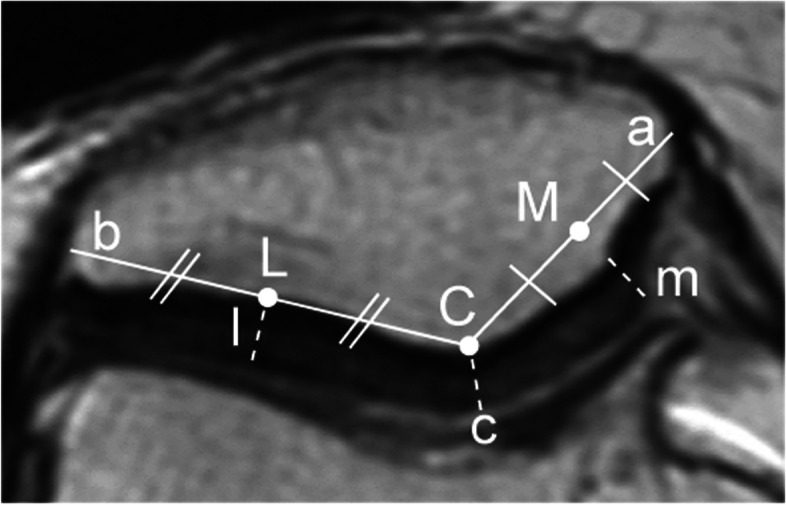
Method of measuring patellar cartilage thickness using an axial single MRI image [a] is a line from the medial edge of the patella to the central edge of the patella; [b] is a line from the lateral edge of the patella to the central edge of the patella; [C] is the central edge of the patella; [M] is the midpoint in line [a]; [L] is the midpoint in line [b]. Dotted lines [c], [m], and [l] represent the cartilage thickness in the central, medial, and lateral facets, respectively. MRI, magnetic resonance imaging

The patellar cartilage thickness was measured according to the following steps: (1) a line, [a], was drawn from the medial edge of the patella to the central edge of the patella; (2) a line, [b], was drawn from the lateral edge of the patella to the central edge of the patella; (3) the midpoint of line [a] was labelled [M], the midpoint of line [b] was labelled [L], (4) and the central edge of the patella was labelled [C]; (5) cartilage thicknesses in the central, medial, and lateral regions were calculated at [C], [M], and [L] (Fig. 1). The dotted lines [c], [m], and [l] are the cartilage thicknesses in the central, medial, and lateral facets, respectively. To standardise the variation in patellar thickness among patients, the percent cartilage thickness was calculated.

### Statistical analysis

All data are presented as mean ± standard deviation. A commercially available software program (GraphPad Software, Inc., San Diego, CA, USA) was used for the statistical calculations. An a priori power analysis was performed. In a study by Kumahashi et al. [18], the difference between preoperative TKA JOA scores and those after 1 year was 25, and the standard deviation was estimated to be 20. Based on this result, a sample size of 18 was calculated to have a 95% power to test our hypotheses. The paired *t-*test was used to assess radiological differences between before TKA and 5 years after TKA. Repeated-measures analysis of variance was used to assess clinical differences between before TKA and 1 and 5 years after TKA, to compare the amount of change in cartilage thickness preoperatively and 5 years postoperatively in the central, medial, and lateral cartilages after TKA, and to assess the percentage cartilage thickness and the cartilage thickness of the central, medial, and lateral facets over a 5-year period. The association between changes in patellar thickness in the central, medial, and lateral facets and functional knee score 5 years after TKA was calculated using Pearson correlation. The significance level was set at *P* = 0.05.

## Results

### Overall clinical and radiological outcomes

The component position was appropriate for all the cases (Table 2). The JOA score and the Oxford knee score significantly improved 1 year postoperatively, but these improved scores significantly worsened 5 years postoperatively (Table 3). The rate of AKP was 18.7%, 5 years postoperatively. The mean lateral femorotibial angle changed significantly from 183.1. to 173.3°. The mean MA shifted to a point 47.9% lateral to the medial edge of the tibial plateau. The Insall–Salvati index and Caton–Deschamps index did not significantly change preoperatively and 5 years postoperatively. The mean tilting angle and congruence angle did not significantly change preoperatively and 5 years postoperatively. The mean lateral shift ratio significantly increased from 7.1% to 14.6%. The CTA twist angle did not show significant change between before surgery and 5 years postoperatively (Table 4).

**Table 2:** Patients’ Knee Society grading scores

Knee Society grading scale	
α	98.0 (3.2)
β	89.8 (2.6)
γ	3.9 (2.7)
δ	85.5 (3.0)

Data are reported as mean (standard deviation)

**Table 3:** Comparison of preoperative, 1-year postoperative, and 5-year postoperative clinical and radiological outcomes

	Preoperative	1-year postoperative	5-year postoperative	***P***-value
ROM (degree)				
Extension	−6.2 (10.6)	−1.6 (5.4)	− 1.78 (5.4)	**0.002** ^a^ (Pre vs 1-year post: **0.005** ^b^, pre vs 5-years post: **0.008** ^b^)
Flexion	125.9 (14.7)	115.8 (10.2)	113.5 (8.2)	**< 0.0001** ^a^ (Pre vs 1-year post: **< 0.0001** ^b^, pre vs 5-years post: **< 0.0001** ^b^, 1-year post vs 5-years post: **0.007** ^b^)
JOA score (points)	52.3 (10.0)	84.8 (5.0)	77.5 (7.4)	**< 0.0001** ^a^ (Pre vs 1-year post: **< 0.0001** ^b^, pre vs 5-years post: **< 0.0001** ^b^, 1-year post vs 5-years post: **< 0.0001** ^b^)
Oxford knee score (points)	17.7 (4.4)	35.0 (2.3)	32.3 (3.6)	**< 0.0001** ^a^ (Pre vs 1-year post: **< 0.0001** ^b^, pre vs 5-years post: **< 0.0001** ^b^, 1-year post vs 5-years post: **< 0.0001** ^b^)

Data are reported as mean (standard deviation), unless otherwise indicated. Bolded *P*-values indicate statistical significance (*P* < .05)

*JOA score* Japanese Orthopedic Association score for osteoarthritic knee, *ROM* range of motion

^a^One-way analysis of variance

^b^Significant difference between preoperative, 1-year postoperative, and 5-year postoperative groups (post hoc Tukey–Kramer test)

**Table 4:** Comparisons of preoperative and 5-year postoperative clinical and radiological outcomes

	Preoperative	5-year postoperative	*P*-value
FTA (°)	184.0 (4.4)	173.6 (3.4)	**< 0.0001**
MA (%)	1.8 (18.4)	48.3 (12.5)	**< 0.0001**
Insall–Salvati ratio	1.1 (0.13)	1.0 (0.15)	0.1521
Caton–Deschamps index	1.0 (0.11)	0.98 (0.11)	0.4606
Tilting angle (°)	5.5 (3.4)	4.6 (4.4)	0.1583
Congruence angle (°)	9.3 (9.0)	9.3 (10.0)	0.6018
Lateral shift ratio (%)	7.8 (5.4)	14.8 (6.0)	**< 0.0001**
Condylar twist angle (°)	2.7 (1.8)	2.8 (1.7)	0.9525

Data are reported as the mean (standard deviation), unless otherwise indicated. Bolded P-values indicate statistical significance (*P* < .05)

Significance levels with Student’s *t*-test

*FTA* lateral femorotibial angle, *MA* mechanical axis

### Longitudinal analyses

**Fig. 2:**
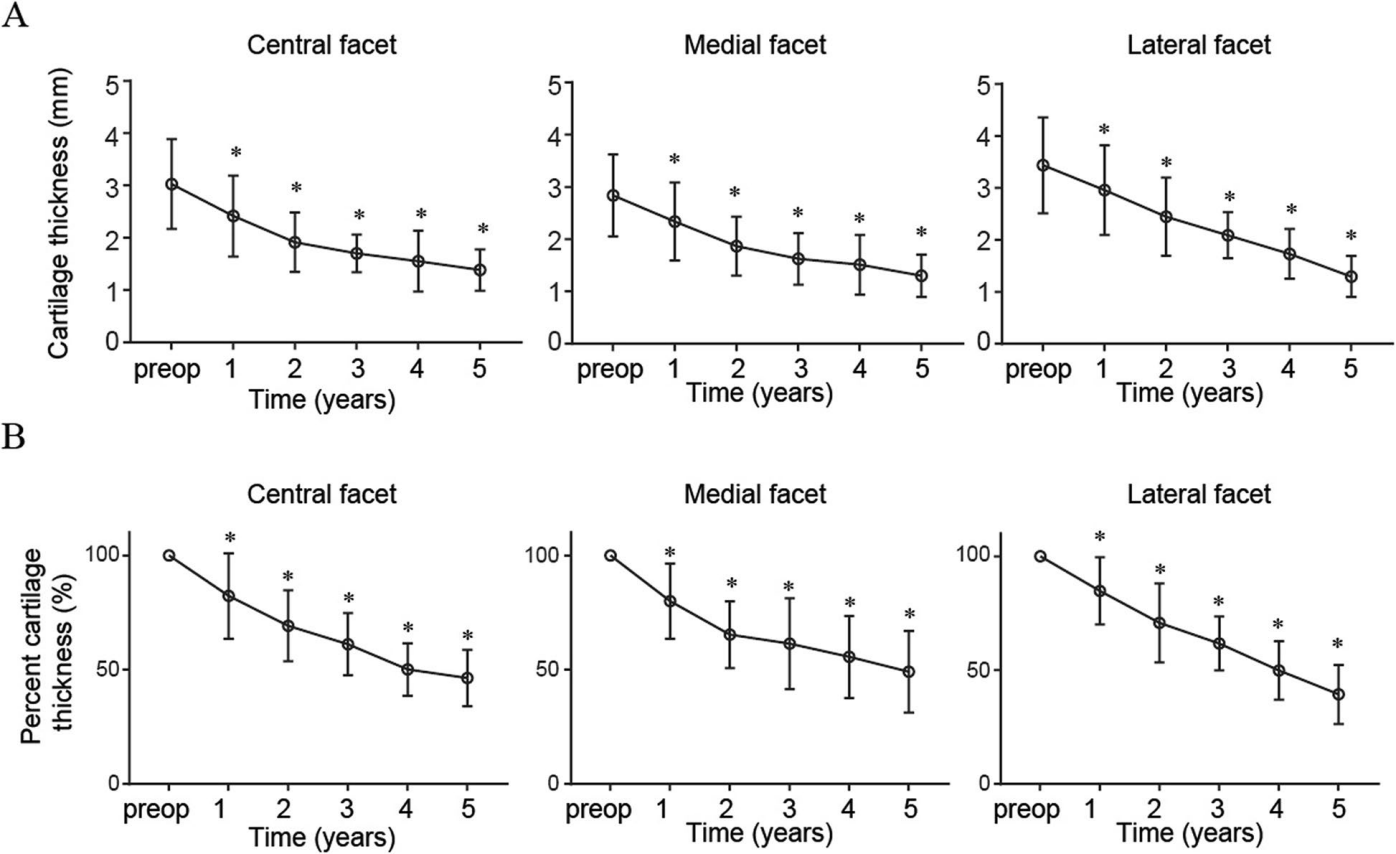
Longitudinal analyses of the percent cartilage thickness and cartilage thickness over a 5-year period. Longitudinal evaluation of changes in patellar cartilage thickness in patients undergoing non-patellar resurfacing total knee arthroplasty. *P < 0.05, versus preoperative value

**Fig. 3:**
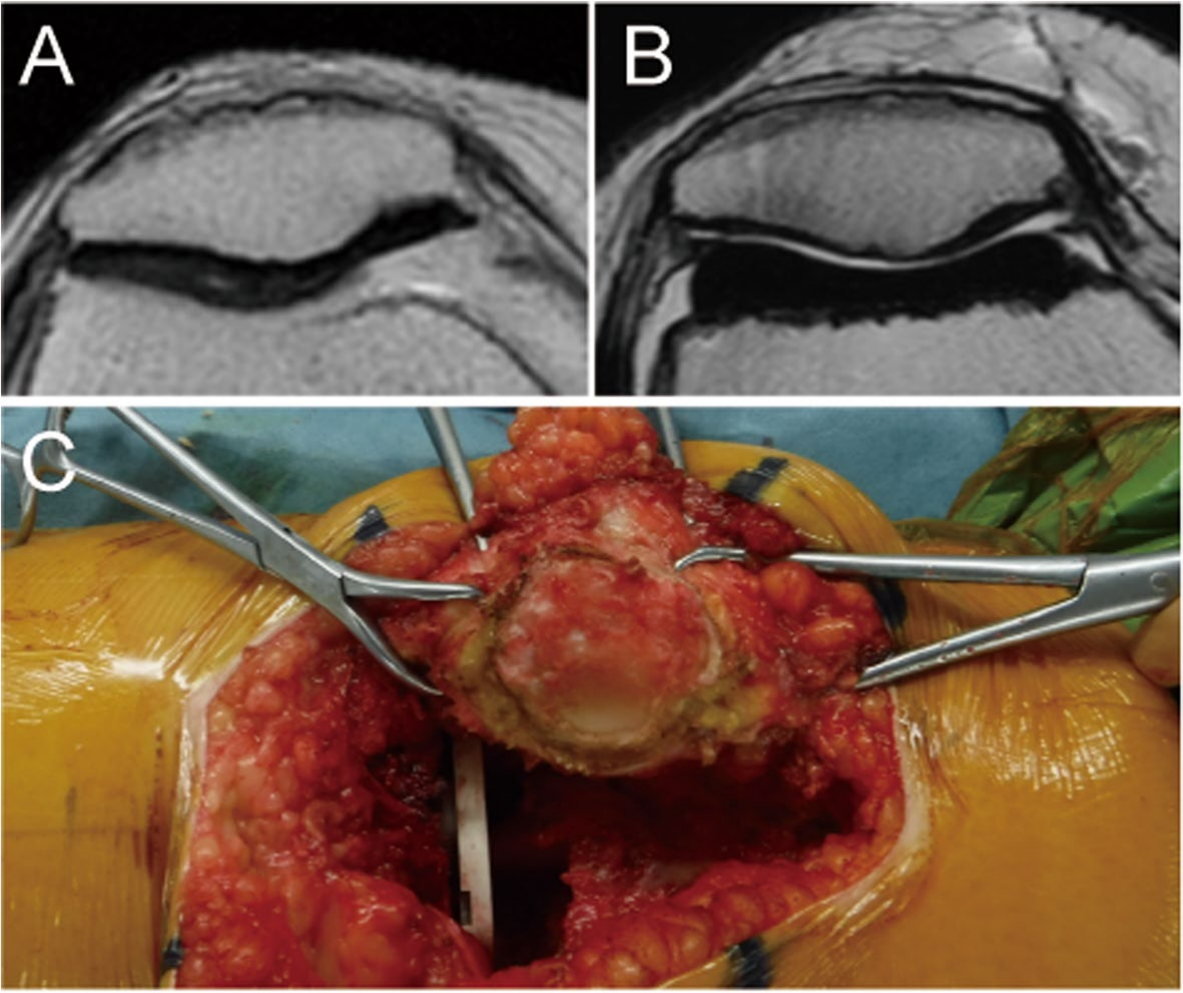
The patient underwent conversion to patellar resurfacing after non-patellar resurfacing TKA. **A** MRI before surgery (**B**) MRI after non-patella resurfacing TKA; the patella thickness is decreased compared to the thickness before TKA (**C**) Intraoperative view of the patellar cartilage reveals excessive articular cartilage wear of the patella in patients undergoing non-patella resurfacing total knee arthroplasty. TKA, total knee arthroplasty; MRI, magnetic resonance imaging

Longitudinal data showed that the percentage cartilage thickness and cartilage thickness of the central, medial, and lateral facets thinned gradually over the 5-year period (Fig. 2A and B). The percent cartilage thickness and cartilage thickness of the central, medial, and lateral facets 5 years postoperatively were significantly decreased compared to the preoperative values (Table 5). The degree of change in cartilage thickness in the lateral facet was significantly decreased compared to that in other facets (central vs. lateral < 0.0001, medial vs. lateral 0.0005) (Table 6). Four patients underwent conversion to patellar resurfacing due to AKP after non-patellar resurfacing TKA (Fig. 3).

**Table 5:** Comparisons of the preoperative and 5-year postoperative patellar cartilage thickness and percentage cartilage thickness

	Preoperative		5-year postoperative		*P*-value	
	Thickness	% thickness	Thickness	% thickness	Thickness	% thickness
Central facet	2.87 (0.74)	100	1.30 (0.41)	46.3 (12.3)	**< 0.0001**	**< 0.0001**
Medial facet	3.03 (0.86)	100	1.38 (0.39)	49.1 (17.8)	**< 0.0001**	**< 0.0001**
Lateral facet	3.44 (0.92)	100	1.29 (0.39)	39.3 (12.9)	**< 0.0001**	**< 0.0001**

Data are reported as the mean (standard deviation), unless otherwise indicated. Bolded P-values indicate statistical significance (*P* < .05)

Significance levels with Student’s *t*-test

**Table 6:** Comparisons of the degree of cartilage thickness change in the central, medial, and lateral facets

	Central facet	Medial facet	Lateral facet	***P***-value
Amount of change in cartilage thickness	1.57(0.63)	1.64 (0.82)	2.14 (0.83)	**< 0.0001** ^a^ (Central vs Lateral **< 0.0001** ^b^, Medial vs Lateral **0.0005** ^b^)

Data are reported as mean (standard deviation) unless otherwise indicated. Bolded *P*-values indicate statistical significance (*P* < .05)

^a^One-way analysis of variance

^b^Significant difference between groups central and lateral facet, medial and lateral facet (post hoc Tukey–Kramer test)

### Correlations between the cartilage thickness in the patella (central, medial, and lateral facets) and functional knee score (JOA score and Oxford knee score)

**Fig. 4:**
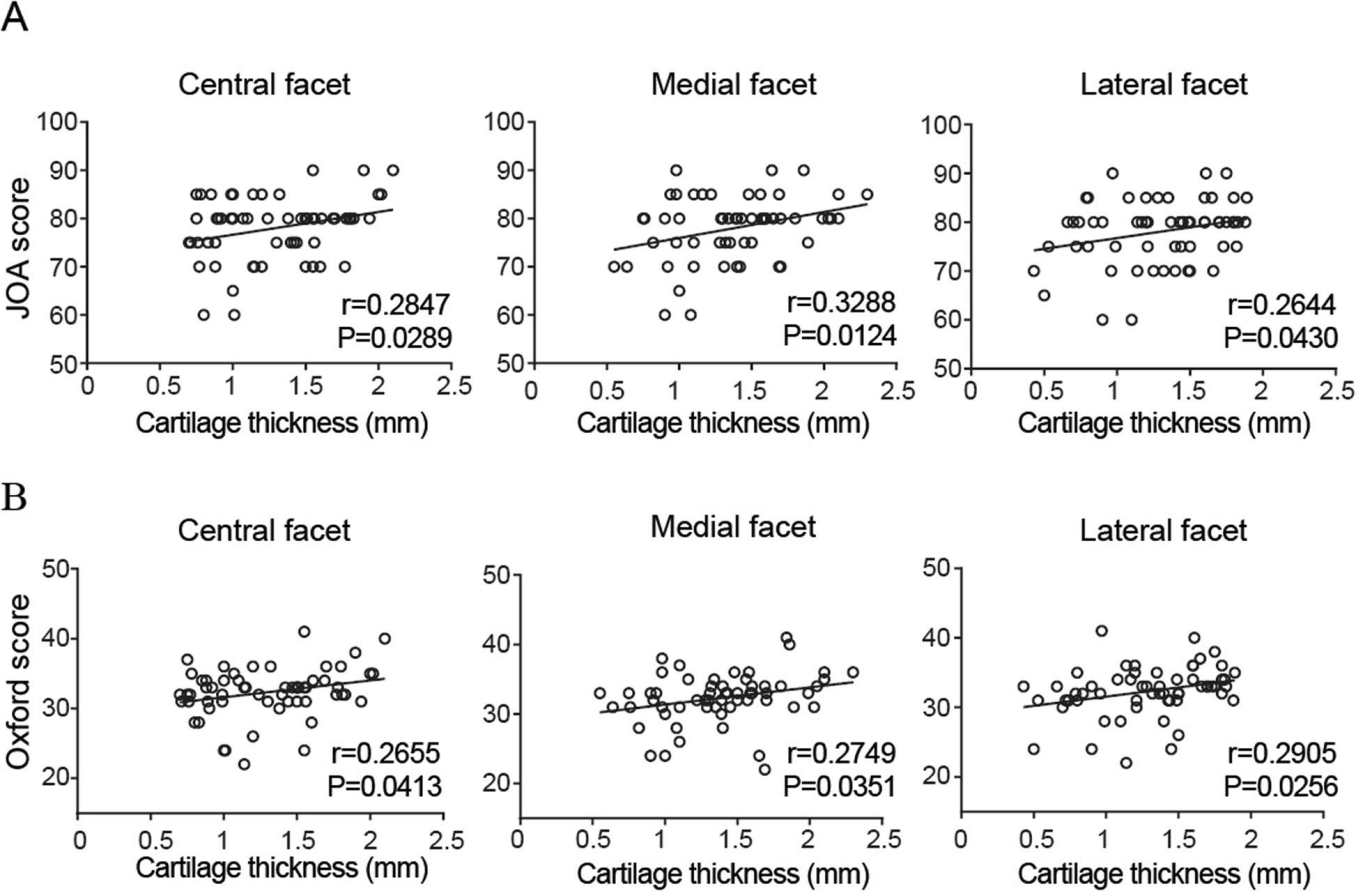
Correlations between cartilage thickness and functional knee score. Correlation analyses between cartilage thickness in the patella (central, medial, and lateral facets) and (**A**) JOA score and (**B**) Oxford knee score 5 years after total knee arthroplasty. JOA score, Japanese Orthopedic Association score

**Fig. 5:**
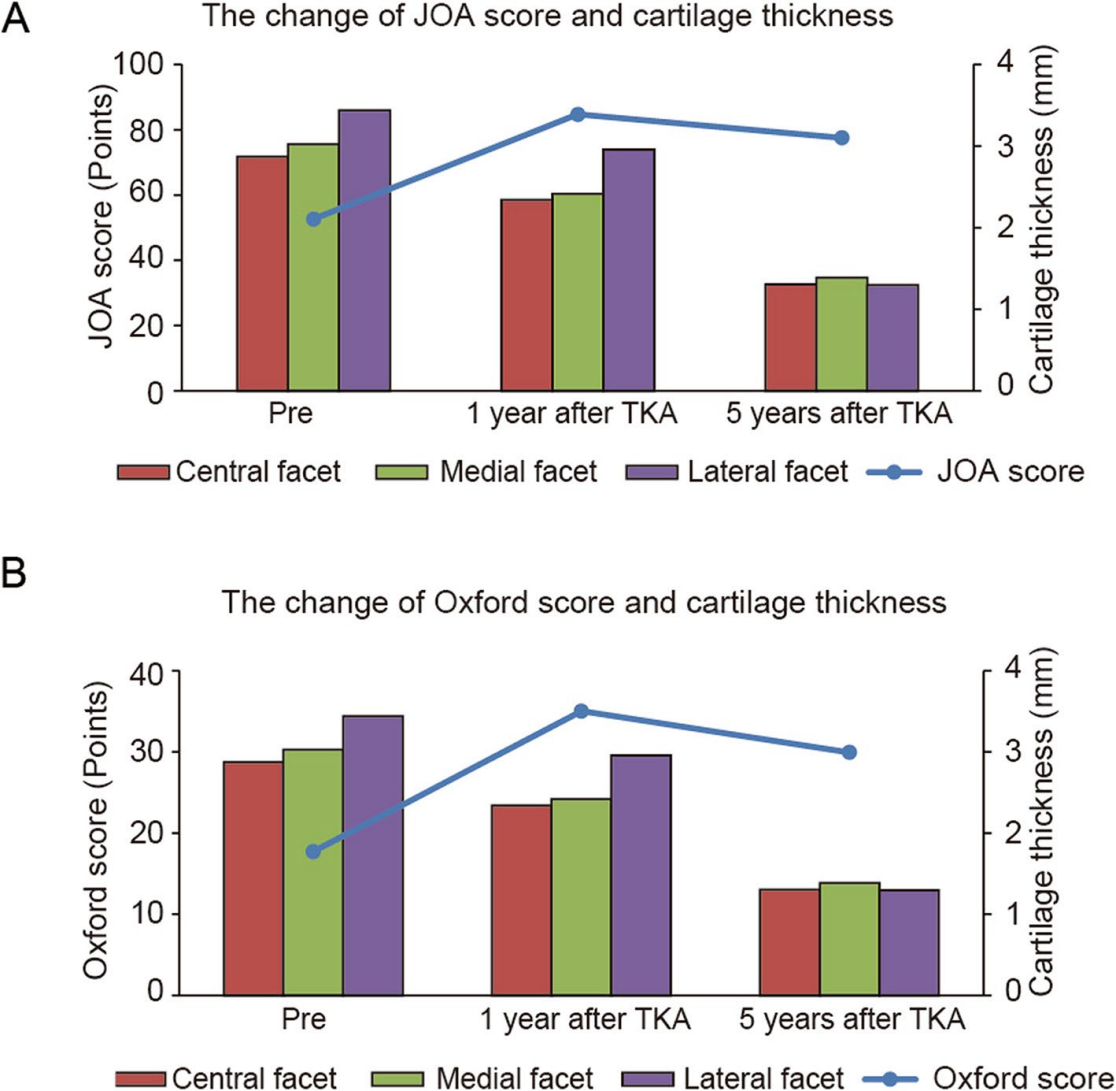
Comparative changes in cartilage thickness and functional knee score before surgery and at 1 and 5 years after TKA. The change in cartilage thickness of the patella (central, medial, and lateral facets) and (**A**) JOA score and (**B**) Oxford knee score before surgery and at 1 and 5 years after TKA. JOA score, Japanese Orthopedic Association score; TKA, total knee arthroplasty

A significant association was observed between the cartilage thickness and functional knee scores 5 years after TKA. Cartilage thickness in the central, medial, and lateral facets was significantly associated with JOA scores 5 years after TKA (Fig. 4A). Cartilage thickness in the central, medial, and lateral facets was significantly associated with Oxford knee score 5 years after TKA (Fig. 4B). A positive correlation suggested a PF joint problem in decreasing patellar cartilage. In addition, both the knee scores and cartilage thickness at 5 years after surgery were lower than those at 1 year after surgery (Fig. 5).

## Discussion

The most important finding of the present study was that the cartilage thickness in the patella was reduced to less than half its preoperative level within 5 years after non-patellar resurfacing TKA despite the use of a zirconia ceramic implant with high biocompatibility. Furthermore, the functional knee score significantly worsened with a reduction in cartilage thickness in the patella. These results suggest that non-patellar resurfacing TKA, despite the use of a zirconia ceramic implant, might cause PF joint problems.

Although there have been many reports that have compared the clinical outcomes and complication rates between patellar resurfacing and non-patellar resurfacing in TKA [6, 9, 19, 24, 25], to our knowledge, there are no reports on the longitudinal observation of changes in patellar cartilage thickness after non-patellar resurfacing TKA. Assessment of patellar cartilage thickness using MRI has high technical validity and is appropriate [8]. Previously, Kumahashi et al. reported that dGEMRIC allowed the quantitative evaluation of the patellar cartilage after TKA [18]. In that study, the postoperative dGEMRIC values of the outer medial half of the superficial zone of the patellar cartilage in the non-resurfacing group were significantly decreased compared to the preoperative values, and the postoperative cartilage thickness of the outer zone of the patella was significantly thinner than the preoperative thickness. However, the study by Kumahashi et al. showed changes in the thickness of the patellar cartilage only 1 year postoperatively and did not evaluate longitudinal changes. In contrast, our data included longitudinal measurements of the thickness of the patellar cartilage over a 5-year period, and clearly suggest that non-patellar resurfacing TKA may result in a decrease in the thickness of the patellar cartilage despite using a zirconia ceramic implant. The strength of our study was the longitudinal observation of changes in patellar cartilage thickness over a 5-year period after non-patellar resurfacing TKA.

In our study, we used a zirconia ceramic implant (LFA®) with high biocompatibility. Nevertheless, cartilage thickness in the patella was reduced to less than half of the preoperative value within 5 years after non-patellar resurfacing TKA. Decreasing patellar cartilage thickness mainly depends on the increasing pressure on the PF joint. The cause of increasing pressure on the PF joint is dependent on body mass index [15], implant design, implant position, radiological factors including patella height [20], and patella tracking course. Regarding implant design, LFA®, which was designed to accommodate the non-resurfaced patella, was used in our study. The patella groove design has an anatomical femoral groove angle of approximately 130° [33]. Therefore, it does not seem to be associated with an increase in pressure on the PF joint and implant design. Next, the implant position was appropriate in all cases, and radiological factors, such as patellar height, did not change before and after TKA. Finally, in the patella tracking course, the patella is inclined to shift laterally due to the change in knee alignment from varus to normal, so that the pressure of the PF joint might be changed. This was consistent with the fact that the cartilage thickness of the lateral facet decreased compared to that of the central and medial facets postoperatively. A previous study reported that the cartilage thickness of the lateral zone was significantly thinner postoperatively, and the patella was inclined laterally (lateral shift ratio was 10.8) [23]. The PF joint pressure may be localised and concentrated on the lateral facet of the patella. This finding suggested that the pressure on the PF joint, especially the lateral zone, may have been increased in our study.

A previous systematic review showed that most meta-analyses unanimously reported equivalent results after patellar resurfacing compared to non-resurfacing in terms of functional scores and complication rates; however, an increased risk of reoperation after patellar non-resurfacing was reported [9]. Persistent AKP remains an important clinical issue after non-resurfacing patella TKA [10]. Its exact aetiology remains subtle, and the effects of prosthesis design, surgical technique, the degree of patellar chondromalacia, preoperative AKP, and patellar tracking alteration on the prevalence of postoperative AKP remain undefined [1]. A previous study reported an average AKP incidence of 10% in non-resurfaced cases compared to 3.3% in resurfaced cases [24]. After 5 years of follow-up, 5% of patients in that study had persistent AKP in the resurfaced side compared to 23% of patients who complained of such pain in the non-resurfaced side. The overall incidence of AKP was higher in patients who underwent TKA and had a non-resurfaced patella than in those who had a resurfaced patella. In our study, the incidence of AKP 5 years postoperatively was 18.7%, which was similar to previously reported data. However, it can be clinically difficult to determine the cause of AKP in a patient without patellar resurfacing, as many other pathologies can have similar presentations (subclinical infection, patellar maltracking, mid-flexion instability, and component malrotation). We should also interpret these results with caution because the use of different scoring systems has resulted in variations in objective AKP assessment and has contributed to the observed heterogeneity. Moreover, the rate of reoperation for patellar non-resurfacing was the main concern for the investigation. In most previous studies, a lower risk for reoperation was reported after patellar resurfacing compared to that after non-resurfacing [13, 19, 25]. In contrast, Parvizi et al. [23] reported no significant difference in the re-intervention rate between the resurfaced and non-resurfaced patella. The cumulative percentage revision rate for patellar resurfacing after non-resurfacing patella TKA was reported to be 10–15% after primary TKA within a 5–10-year follow-up period [2, 19]. The revision rate in our study (6.7%) was lower than that in previous studies. Furthermore, patients’ activity levels must be considered. The mean age of our patients (72.2 years) was higher than that of patients in other studies (the mean age ranged from 60 to 70 years) [2, 19]. Therefore, the activity level of patients in our study may have been lower; hence, close attention had to be paid to our patients to avoid overloading the implants.

We must consider the effect of decreasing cartilage thickness in the patella on clinical outcomes. This study demonstrated a correlation between decreasing cartilage thickness in the patella and functional knee scores. Decreasing cartilage thickness in the patella can cause PF joint problems. Decreasing cartilage thickness is caused by vigorous physical activities, such as jogging, squatting, and climbing stairs, that subjects the knee to repeated stress. It can also be caused by abnormal tracking of the patella in the trochlear groove. Hence, we must consider that the scoring system is not specific for PF disorders. The total JOA score for osteoarthritic knees was a total of 100 points: pain and walking, 30 points; pain and stairs up and down, 25 points; range of motion, 35 points; and swelling, 10 points [32]. The PF joint problem accounts for 25% of the total points. There is a possibility that another factor is related to worsening functional knee score; hence, it may be difficult to determine whether decreasing cartilage thickness in the patella leads to degraded clinical outcomes. To our knowledge, the Kujala score is a specific scoring system for PF disorders to evaluate subjective symptoms and functional limitations in PF disorders [17]. However, it includes high-load activities, such as squatting, running, and jumping. Therefore, it is not suitable to evaluate the PF joint outcome after TKA in elderly patients. Although it was necessary to assess the correlation between decreasing cartilage thickness in the patella and the functional knee score precisely, it might be inadequate to use only the current scoring system.

This study has several limitations. First, the number of patients was small due to the limited criteria for indication in this study. Therefore, further studies with larger sample sizes are required to verify the findings of this study. Second, this study lacked a control, which could have been a non-resurfaced patella CoCr implant in TKA. We did not evaluate the difference between the effect of ceramic implants and that of metal implants on cartilage thickness in non-resurfaced patella in TKA. However, in case of metal implants, it is difficult to assess the cartilage thickness accurately using MRI due to metal artefacts and make comparisons between the ceramic and metal implants. Third, we evaluated only one slice of the patella using MRI. We selected an axial MR image slice, which was the centre of the patella, to evaluate patellar thickness. Therefore, errors in image slice selection may occur and influence the measurement of patellar cartilage thickness. Fourth, we evaluated the implant positioning using conventional X-rays and 2D-CT. The most accurate method to evaluate implant positioning is 3D-CT [12]; however, we did not perform a full limb CT in this series. We could not rule out the effect of rotational or sagittal malalignment of the femoral component precisely on clinical outcomes. Finally, we did not compare and evaluate the natural course of degeneration of the patellar cartilage in intact knees of elderly patients with annual MRI.

## Conclusion

The cartilage thickness in the patella was reduced to less than half of the preoperative level within 5 years after non-patellar resurfacing TKA. This would lead to clinical symptoms of AKP and conversion to patellar resurfacing.

## Abbreviations

AKP: Anterior knee pain
CTA: Condylar twist angle
CT: Computed tomography
dGEMRIC: Delayed gadolinium-enhanced magnetic resonance imaging of cartilage
JOA: Japanese Orthopedic Association
MRI: Magnetic resonance imaging
OA: Osteoarthritis
PF: Patellofemoral
TKA: Total knee arthroplasty

## References

[1] Arbuthnot JE, McNicholas MJ, McGurty DW, Rowley DI (2004). Total knee replacement and patellofemoral pain. Surgeon.

[2] Beaupre L, Secretan C, Johnston DW, Lavoie G (2012). A randomized controlled trial comparing patellar retention versus patellar resurfacing in primary total knee arthroplasty: 5-10 year follow-up. BMC Res Notes.

[3] Burnett RS, Haydon CM, Rorabeck CH, Bourne RB (2004). Patella resurfacing versus nonresurfacing in total knee arthroplasty: results of a randomized controlled clinical trial at a minimum of 10 years' followup. Clin Orthop Relat Res.

[4] Chen K, Dai X, Li L, Chen Z, Cui H, Lv S (2021). Patellar resurfacing versus nonresurfacing in total knee arthroplasty: an updated meta-analysis of randomized controlled trials. J Orthop Surg Res.

[5] Dawson J, Fitzpatrick R, Murray D, Carr A (1998). Questionnaire on the perceptions of patients about total knee replacement. J Bone Joint Surg Br.

[6] Deroche E, Batailler C, Swan J, Sappey-Marinier E, Neyret P, Servien E et al (2021) No difference between resurfaced and non-resurfaced patellae with a modern prosthesis design: a prospective randomized study of 250 total knee arthroplasties. Knee Surg Sports Traumatol Arthrosc. 10.1007/s00167-021-06521-y 10.1007/s00167-021-06521-y 33661323

[7] Ewald FC (1989). The knee society total knee arthroplasty roentgenographic evaluation and scoring system. Clin Orthop Relat Res.

[8] Graichen H, von Eisenhart-Rothe R, Vogl T, Englmeier KH, Eckstein F (2004). Quantitative assessment of cartilage status in osteoarthritis by quantitative magnetic resonance imaging: technical validation for use in analysis of cartilage volume and further morphologic parameters. Arthritis Rheum.

[9] Grassi A, Compagnoni R, Ferrua P, Zaffagnini S, Berruto M, Samuelsson K (2018). Patellar resurfacing versus patellar retention in primary total knee arthroplasty: a systematic review of overlapping meta-analyses. Knee Surg Sports Traumatol Arthrosc.

[10] Ha C, Wang B, Li W, Sun K, Wang D, Li Q (2019). Resurfacing versus not-resurfacing the patella in one-stage bilateral total knee arthroplasty: a prospective randomized clinical trial. Int Orthop.

[11] Hayter CL, Koff MF, Shah P, Koch KM, Miller TT, Potter HG (2011). MRI after arthroplasty: comparison of MAVRIC and conventional fast spin-echo techniques. AJR Am J Roentgenol.

[12] Hirschmann MT, Konala P, Amsler F, Iranpour F, Friederich NF, Cobb JP (2011). The position and orientation of total knee replacement components: a comparison of conventional radiographs, transverse 2D-CT slices and 3D-CT reconstruction. J Bone Joint Surg Br.

[13] Hunt LP, Matharu GS, Blom AW, Howard PW, Wilkinson JM, Whitehouse MR (2021). Patellar resurfacing during primary total knee replacement is associated with a lower risk of revision surgery. Bone Joint J.

[14] Insall J, Salvati E (1971). Patella position in the normal knee joint. Radiology.

[15] Koff MF, Amrami KK, Kaufman KR (2007). Clinical evaluation of T2 values of patellar cartilage in patients with osteoarthritis. Osteoarthr Cartil.

[16] Kolind SH, MacKay AL, Munk PL, Xiang QS (2004). Quantitative evaluation of metal artifact reduction techniques. J Magn Reson Imaging.

[17] Kujala UM, Jaakkola LH, Koskinen SK, Taimela S, Hurme M, Nelimarkka O (1993). Scoring of patellofemoral disorders. Arthroscopy.

[18] Kumahashi N, Tadenuma T, Kuwata S, Fukuba E, Uchio Y (2013). A longitudinal study of the quantitative evaluation of patella cartilage after total knee replacement by delayed gadolinium-enhanced magnetic resonance imaging of cartilage (dGEMRIC) and T2 mapping at 3.0 T: preliminary results. Osteoarthr Cartil.

[19] Li S, Chen Y, Su W, Zhao J, He S, Luo X (2011). Systematic review of patellar resurfacing in total knee arthroplasty. Int Orthop.

[20] Luyckx T, Didden K, Vandenneucker H, Labey L, Innocenti B, Bellemans J (2009). Is there a biomechanical explanation for anterior knee pain in patients with patella Alta?: influence of patellar height on patellofemoral contact force, contact area and contact pressure. J Bone Joint Surg Br.

[21] Nizard RS, Biau D, Porcher R, Ravaud P, Bizot P, Hannouche D et al (2005) A meta-analysis of patellar replacement in total knee arthroplasty. Clin Orthop Relat Res. 10.1097/01.blo.0000150348.17123.7f196-203 10.1097/01.blo.0000150348.17123.7f 15738822

[22] Pakos EE, Ntzani EE, Trikalinos TA (2005). Patellar resurfacing in total knee arthroplasty. A meta-analysis. J Bone Joint Surg Am.

[23] Parvizi J, Rapuri VR, Saleh KJ, Kuskowski MA, Sharkey PF, Mont MA (2005). Failure to resurface the patella during total knee arthroplasty may result in more knee pain and secondary surgery. Clin Orthop Relat Res.

[24] Patel K, Raut V (2011). Patella in total knee arthroplasty: to resurface or not to--a cohort study of staged bilateral total knee arthroplasty. Int Orthop.

[25] Pavlou G, Meyer C, Leonidou A, As-Sultany M, West R, Tsiridis E (2011). Patellar resurfacing in total knee arthroplasty: does design matter? A meta-analysis of 7075 cases. J Bone Joint Surg Am.

[26] Pilling RW, Moulder E, Allgar V, Messner J, Sun Z, Mohsen A (2012). Patellar resurfacing in primary total knee replacement: a meta-analysis. J Bone Joint Surg Am.

[27] Ranawat CS (2002). History of total knee replacement. J South Orthop Assoc.

[28] Sasaki T, Yagi T (1986). Subluxation of the patella. Investigation by computerized tomography. Int Orthop.

[29] Smith AJ, Wood DJ, Li MG (2008). Total knee replacement with and without patellar resurfacing: a prospective, randomised trial using the profix total knee system. J Bone Joint Surg Br.

[30] Waters TS, Bentley G (2003). Patellar resurfacing in total knee arthroplasty. A prospective, randomized study. J Bone Joint Surg Am.

[31] Wood DJ, Smith AJ, Collopy D, White B, Brankov B, Bulsara MK (2002). Patellar resurfacing in total knee arthroplasty: a prospective, randomized trial. J Bone Joint Surg Am.

[32] Yasuda K, Majima T, Tsuchida T, Kaneda K (1992). A ten- to 15-year follow-up observation of high tibial osteotomy in medial compartment osteoarthrosis. Clin Orthop Relat Res.

[33] Yasuda K, Miyagi N, Kaneda K (1993). Low friction total knee arthroplasty with the alumina ceramic condylar prosthesis. Bull Hosp Jt Dis.

